# Synthesis of zinc oxide nanoparticles using methanol propolis extract (Pro-ZnO NPs) as antidiabetic and antioxidant

**DOI:** 10.1371/journal.pone.0289125

**Published:** 2023-07-25

**Authors:** Dwi Ajeng P. D., Dyna Ratnasari Plashintania, Rindia M. Putri, Indra Wibowo, Yusrin Ramli, Sabrina Herdianto, Antonius Indarto

**Affiliations:** 1 School of Life Sciences and Technology, Institut Teknologi Bandung, Bandung, Indonesia; 2 Biochemistry Research Division, Faculty of Mathematics and Natural Sciences, Institut Teknologi Bandung, Bandung, Indonesia; 3 Graduate School of Science and Technology, Hirosaki University, Hirosaki, Japan; 4 Department of Chemical Engineering, Institut Teknologi Bandung, Bandung, Indonesia; 5 Department of Bioenergy Engineering and Chemurgy, Institut Teknologi Bandung, Bandung, Indonesia; University of Botswana, BOTSWANA

## Abstract

In recent times, the overall health of individuals has been declining due to unhealthy lifestyles, leading to various diseases, including diabetes. To address this issue, antidiabetic and antioxidant agents are required to back-up human well-being. Zinc oxide (ZnO) is one such substance known for its antidiabetic and antioxidant effects. To enhance its capability and effectiveness, propolis was utilized to synthesize zinc oxide nanoparticles (Pro-ZnO NPs). The objective of this study was to synthesize Pro-ZnO NPs and assess their performance by conducting inhibition assays against α-amylase and α-glucosidase enzymes, as well as a 2,2-diphenyl-1-picrylhydrazyl (DPPH) free radical scavenging assay. The results showed that Pro-ZnO NPs were formed in a hexagonal wurtzite structure, with particle sizes ranging from 30 to 50 nm and an absorption band observed at 341 nm. The stability, chemical properties, and crystallography of Pro-ZnO NPs were also thoroughly examined using appropriate methods. The Pro-ZnO NPs demonstrated significant inhibitory effects against α-amylase and α-glucosidase enzymes, with inhibition rates reaching 69.52% and 73.78%, respectively, whereas the antioxidant activity was as high as 70.76%. Consequently, with their high inhibition rates, the Pro-ZnO NPs demonstrate the potential to be employed as a natural agent for combating diabetes and promoting antioxidant effects.

## Introduction

In the 21^st^ century, human wellness is getting worse and worse, as witnessed by many critical diseases as well as diabetes [1, 2]. On the one hand, the number of diabetic people is estimated will increase to 628.6 million people by 2045 [1]. Due to this condition, novel prevention is needed such as utilizing advanced technology. One of the promising technology is nanotechnology since it has grown rapidly in the last decades and can be used widely for human prosperities, including human health [3]. Shortly, nanotechnology can create tiny particles, namely, nanoparticles [4] that are vastly used in medical fields because of their advantages of moving rapidly and specifically in the human body [5]. Among diverse types of nanoparticles, metal oxide-based nanoparticles have gained much attention due to their various catalytic and electronic properties.

One of the metal-oxide nanoparticles that can be chosen is zinc oxide (ZnO) because it has unique properties such as semi-conductive, a wide range of radiation absorption, piezoelectric, pyroelectric, high catalytic, and good stability [6]. In addition, ZnO possesses non-toxic properties and has been listed as “Generally recognized as safe” (GRAS) by the US Food and Drug Administration [7]; furthermore, zinc oxide nanoparticles (ZnO-NPs) are also known to have numerous bioactivities including antidiabetic and antioxidant [8–11]. Importantly, ZnO has high availability and is comparable with silicon dioxide (SiO_2_) and tin oxide (TiO_2_).

Generally, ZnO NPs can be synthesized using the top-down methods (mechanical processes) by breaking up large materials into small nano-sized particles and the bottom-up methods (chemical processes) by forming nanoparticles from molecular or ionic precursors [12]. Nevertheless, these two methods are considered less environmentally friendly. The mechanical methods usually require to be conducted at high energy as well as high pressure, which might be time-consuming and result in a high capital cost [13, 14]. Meanwhile, the chemical methods involve the use of toxic chemicals to synthesize NPs that might cause unwanted effects on the NPs or by-products generated [15]. To overcome these weaknesses, biological methods have emerged as an alternative to conventional physical and chemical methods.

In biological methods, nanoparticles are synthesized using organic compounds produced by organisms or natural products that act as reducing and stabilizing agents [13]. The biological methods provide several advantages owing to their simple, cost-efficient, non-toxic, and eco-friendly nature [4, 11, 16]. Additionally, there are various primary sources of nanoparticle biosynthesis applications along with bacteria, fungi, plants, and natural products. Among them, synthesized nanoparticles using plant and natural products are more advantageous than those using microorganisms since it provides a simpler handling process and does not require complex treatments such as isolation, culture maintenance, and a series of purification processes [15].

One of the considerations is propolis, a resinous substance collected by bees from several parts of plants, shoots, and exudates mixed with wax compounds and bees’ saliva, which are then used to repair and maintain beehives [17]. Interestingly, the hydroxyl, carbonyl, and amine functional groups contained in propolis can react with the metal ions and reduce their size to a nanometer dimension [18]. In order to extract the valuable components, methanol is designated as the solvent since it has the most effective ability to extract the metabolite compounds contained in propolis compared to other solvents such as water, ethanol, chloroform, dichloromethane, ether, and acetone [19].

In previous studies, ZnO NPs have been scrutinized for the effectiveness on antidiabetic, antioxidant, and antimicrobial which the ZnO NPs were extracted by using plants or microorganisms [7, 9–11, 20–22]. For instance, Sharma et. al. (2022) have reported the effectiveness of ZnO NPs by using *Murraya koenigii* leaf extract as shown by amylase inhibition between 56.2 and 75.3 μg/mL [10]. On the other hand, in 2019, Vinotha et. al. has been reported their work about an antidiabetic property of ZnO NPs which is extracted by using *Costus igneus* as evidenced by the %inhibition of amylase as much as 36% to 82% [7].

To date, the synthesis of ZnO NPs by using propolis is rarely disclosed, albeit propolis has been proven that it can reduce and stabilize silver nanoparticles (AgNP) [13] and gold nanoparticles (AuNP) [23]. Furthermore, propolis also has various bioactivities as well as wound healing, anti-inflammation, antidiabetic, antimicrobial, hepatoprotective, and antioxidants [24]. On the other hand, there are several studies have shown that various types of plant extracts and natural ingredients can be utilized in the biosynthesis process of zinc oxide nanoparticles and produce various shapes and sizes of nanoparticles [7, 25, 26]. However, ZnO NPs synthesis using propolis methanolic extract studies are rarely disclosed.

In short, this study aims to synthesize and characterize zinc oxide nanoparticles using methanol propolis extract (Pro-ZnO NPs) with the biosynthesis method. Also, in this study, the characteristic propolis methanolic and Pro-ZnO NPs were carried out by using Liquid chromatography-mass spectrometry (LCMS), Nuclear magnetic resonance (NMR), UV–visible spectrophotometry (UV-Vis), Transmission Electron Microscopy (TEM), Fourier-transform infrared (FTIR), X-ray diffraction (XRD), and zeta potential analysis. Furthermore, the effectiveness of Pro-ZnO NPs was evaluated by measuring the antidiabetic and antioxidant properties of synthesized Pro-ZnO NP using inhibitory activity against α-amylase and α-glucosidase enzymes and 2,2-diphenyl-1-picrylhydrazyl (DPPH) free radical scavenging assay, respectively.

## Materials and methods

### Materials

The raw material in this research is propolis, which is obtained from Rembang, East Java, Indonesia. Some chemicals were also used, namely, 99.9% methanol (Merck #1.06009.2500), zinc nitrate hexahydrate (Zn(NO_3_)_2_.6H_2_O) (Sigma Aldrich #228737), distilled water, ethanol technical grade 96% (Rofa), DPPH kit assay (2,2-diphenyl-1-picrylhydrazyl, Sigma Aldrich #D9132), and filter paper no. 1 (Sigma Aldrich).

### Experimental instruments and characterization

The product analyses and experimental instruments both for qualitative and quantitative characterizations were conducted by using LCMS/MS (Waters™, US), Furnace box CWF100 (Carbolite Gero, UK), HT7700 TEM (Hitachi, JP), zeta potential analyzer SZ-100 (Horiba, JP), UV-Vis Spectrophotometer UV-1800 (Shimadzu, JP), Thermo Nicolet iS50 FT/IR (Thermo Scientific™, US), D8 Advance XRD (Bruker, US).

### Preparation of propolis extract

The propolis extraction was done via the maceration technique by immersing propolis in 99.9% methanol as a solvent [27]. Initially, propolis was cut into pieces and dried under the sunshine. Then, as much as 100 g of the small pieces of propolis were soaked in 1:10 methanol solvent and macerated at room temperature for 3 days. Then, the filtrate was strained through filter paper no. 1 to eliminate all the remaining residues. On the other hand, the filtrate was then evaporated at 40°C until the brown paste extract was obtained. Lastly, the extract was stored in the refrigerator (4°C) for supplementary analysis.

### Propolis extract and Pro-ZnO NPs characterization and synthesis

The content of propolis extract was determined by qualitative phytochemical assay and LCMS/MS analysis. A qualitative phytochemical assay was conducted to determine the presence of phytochemical constituents contained in the obtained extract. This test was carried out to detect the presence of phenolic, flavonoid, tannin, alkaloid, terpenoid, and steroid by using standard protocols.

The bioactive compounds of propolis methanolic extract were determined by LCMS/MS using Waters™ Acquity UPLC I-Class and XEVO G2-XS QTof equipped with Waters™ silica column (1.8 μm particle size × 2.1 mm diameter × 100 mm length). The column temperature was maintained at 40°C. An injection volume of 1 μL of the propolis extract was used with methanol as a solvent. In the mass spectrometer analysis, positive ion ESI (Electron Spray Ionization) was used as the ion source with a scan range of 100–1200 m/z.

^1^H NMR spectrum of propolis methanolic extract was carried out using DMSO-d_6_ (deuterated dimethyl sulfoxide) and TMS (tetramethylsilane) as reference compounds. The existence of metabolites in propolis methanolic extract was investigated further by comparing the ^1^H NMR spectra and LCMS data and later analyzed using MestReNova software.

The synthesis process of Pro-ZnO NPs refers to the previous method [25] with some modifications. The process was carried out by gradually heating 25 mL of propolis extract. When the temperature reached 60°C, 2.5 g of zinc nitrate hexahydrate (Zn(NO_3_)_2_.6H_2_O) was added to the extract. After that, the compound was stirred continuously, and the temperature was maintained at 60°C for 1 hour. The color change from dark brown to lighter brown indicates a reduction reaction of Zn^2+^ [28]. This compound was then left overnight in an incubator at 60°C until a white precipitate was formed. Then, the precipitate was placed in a crucible cup. Afterward, the paste was burned in a furnace at a temperature of 700°C for 3 hours. Then, the resulting white powder was stored in an airtight container.

Pro-ZnO NPs propolis formed is characterized by a UV-Vis spectrophotometer in order to determine the formation of Pro-ZnO NPs and their stability [26]. The characterization was carried out by UV-Vis spectrophotometer (Shimadzu UV-1900) with a range of 250–600 nm using a quartz cuvette and distilled water as blanks. Observations of the crystal characteristics, morphology, and size of the nanoparticle samples were carried out using HR-TEM (Hitachi H9500) at magnifications of 50 and 100 nm [25]. Zeta potential and the value of the charge between the surface of the particles were measured using a Zetasizer (Horiba SZ-100) [29].

Furthermore, FTIR was used as a confirmation technique for nanoparticle formation and helped identify possible phytochemical molecules involved in the reduction and stabilization of Pro-ZnO NPs. The FTIR spectrum of Pro-ZnO NPs and propolis extracts was recorded using an FTIR Spectrometer (Thermo Scientific Nicolet iS5) with a wavelength of 4000–400 cm^-1^ [25]. Powder samples were tested using an X-Ray Diffractometer (XRD Bruker D8 Advance) with λ = 1.5406 A° operated at 40 kV and 30 mA at a range of 2θ from 30–140° to confirm the presence of ZnO and to characterize particle crystallinity and the crystallite size through the following Debye–Scherrer equation [25].

### Antidiabetic activity of Pro-ZnO NPs

#### Test of inhibitory activity against α-amylase

The antidiabetic activity of propolis and Pro-ZnO NPs extracts were evaluated in vitro by α-amylase inhibition test using the DNSA method [7]. To begin, samples of Pro-ZnO NPs and propolis extract were tested separately at concentrations of 20, 40, 60, 80, and 100 mg/mL and put into test tubes. Later, 500 μL of 0.02 M sodium phosphate buffer (containing 6 mM NaCl, pH 6.9) and 500 μL of α-amylase solution was added to the solution. Then, the solution was incubated at 37°C for 20 minutes. After incubation, 250 μL of 1% starch solution was added to the tube and incubated for another 15 minutes at 37°C. The reaction was terminated by adding 1 mL of dinitrosalicylic acid (DNS) reagent and then incubated in a water bath at 100°C for 10 minutes. The tube was cooled and absorbance or optical density (OD) was measured at 540 nm. The reaction compound without nanoparticle samples and with metformin at various concentrations (20–100 μg /mL) was used as negative and positive controls, respectively. The inhibitory activity of α-amylase was calculated by using Eq (1).


$$\%{Inhibition}_{A}=\frac{A_{A-control}-A_{A-sample}}{A_{A-control}}\times 100\%$$ (1)

Where, %Inhibition_A_: the inhibition percentage of α-amylase or α-glucosidase (%);

A_A-control_: absorbance of the α-amylase or α-glucosidase control solution; and

A_A-sample_: absorbance of the α-amylase or α-glucosidase sample.

### Test of inhibitory activity against α-glucosidase

The α-glucosidase inhibition test was carried out to confirm the inhibitory activity of propolis and Pro-ZnO NPs extracts by referring to the previous method [7]. The tests were carried out using several concentrations of propolis extract, and ZnO with concentrations of 20, 40, 60, 80, and 100 g/mL added separately to a 5 mL test tube. Firstly, as much as 250 μL of 5 mM of a p-nitrophenyl-α-D glucopyranoside solution and 490 μL of 0.1 M phosphate buffer (pH 6.9) were added to a test tube which is containing samples with various concentrations (20–100 g/mL). After the homogeneous solution was pre-incubated for 5 minutes at 37°C, the reaction was started by adding 250 μL of 0.1 α-glucosidase unit and incubated for 20 minutes at 37°C. The reaction was terminated by adding 50 μL of sodium carbonate (0.1 M of Na_2_CO_3_ solution). The activity of α-glucosidase was determined using spectrophotometry by measuring the absorbance at a wavelength of 405 nm. The reaction mixtures without sample and metformin at different concentrations (20–100 μg/mL) were used as negative and positive controls, respectively. The inhibitory activity of α- glucosidase was also calculated by using Eq (1).

### Antioxidant activity of Pro-ZnO NPs

The antioxidant activity of propolis extract and synthesized Pro-ZnO NPs was determined by DPPH free radical scavenging assay as illustrated previously by Vinotha et al. (2019) [7] with several modifications. Approximately 500 μL of 1 M DPPH solution was dissolved with 99.9% methanol in several concentrations (20, 40, 60, 80, and 100 mg/mL) of propolis extract. The mixture of synthesized Pro-ZnO NPs and ascorbic acid as a positive control. The mixture was shaken strongly and incubated for 30 minutes in the dark at room temperature. After incubation, the absorbance of mixtures was determined using a UV-Vis spectrophotometer (UV-1800, Shimadzu) at OD517nm. The percentage of inhibition of propolis extract, Pro-ZnO NPs, and ascorbic acid was measured by using Eq (2).


$$\%{Inhibition}_{D}=\frac{A_{D-control}-A_{D-sample}}{A_{D-control}}\times 100\%$$ (2)

Where, %Inhibition_D_: the inhibition percentage of DPPH(%);

A_D-control_: absorbance of the DPPH control solution; and

A_D-sample_: absorbance of the DPPH sample.

## Results and discussion

### Phytochemical screening of propolis methanolic extract

Based on qualitative phytochemical screening, the obtained propolis methanolic extract consists of various phytochemical compounds including phenolics, tannins, flavonoids, triterpenoids, and alkaloids as shown in Table 1. Factors affecting the composition of bioactive compounds in propolis are the geographical area that correlates with the plant sources, bees type, and time of harvest [30]. The various chemical and bioactive compounds of propolis are considered reduction and stabilization agents for the biosynthesis of metal nanoparticles [29].

**Table 1:** Phytochemical screening of propolis methanolic extract.

No.	Types	Methods	Result
1	Phenolics	Ferric chloride test	+
2	Tannins	Ferric chloride test	+
3	Flavonoids	a. HCl + Mg test	+
		b. H_2_SO_4_ test	+
		c. NaOH test	+
4	Terpenoids	H_2_SO_4_ + anhydride CH_3_COOH test	+
	Steroids		-
5	Alkaloids	a. Dragendorff’s test	+
		b. Wagner’s test	+

### LCMS/MS and ^1^H NMR analysis of propolis methanolic extract

**Fig 1:**
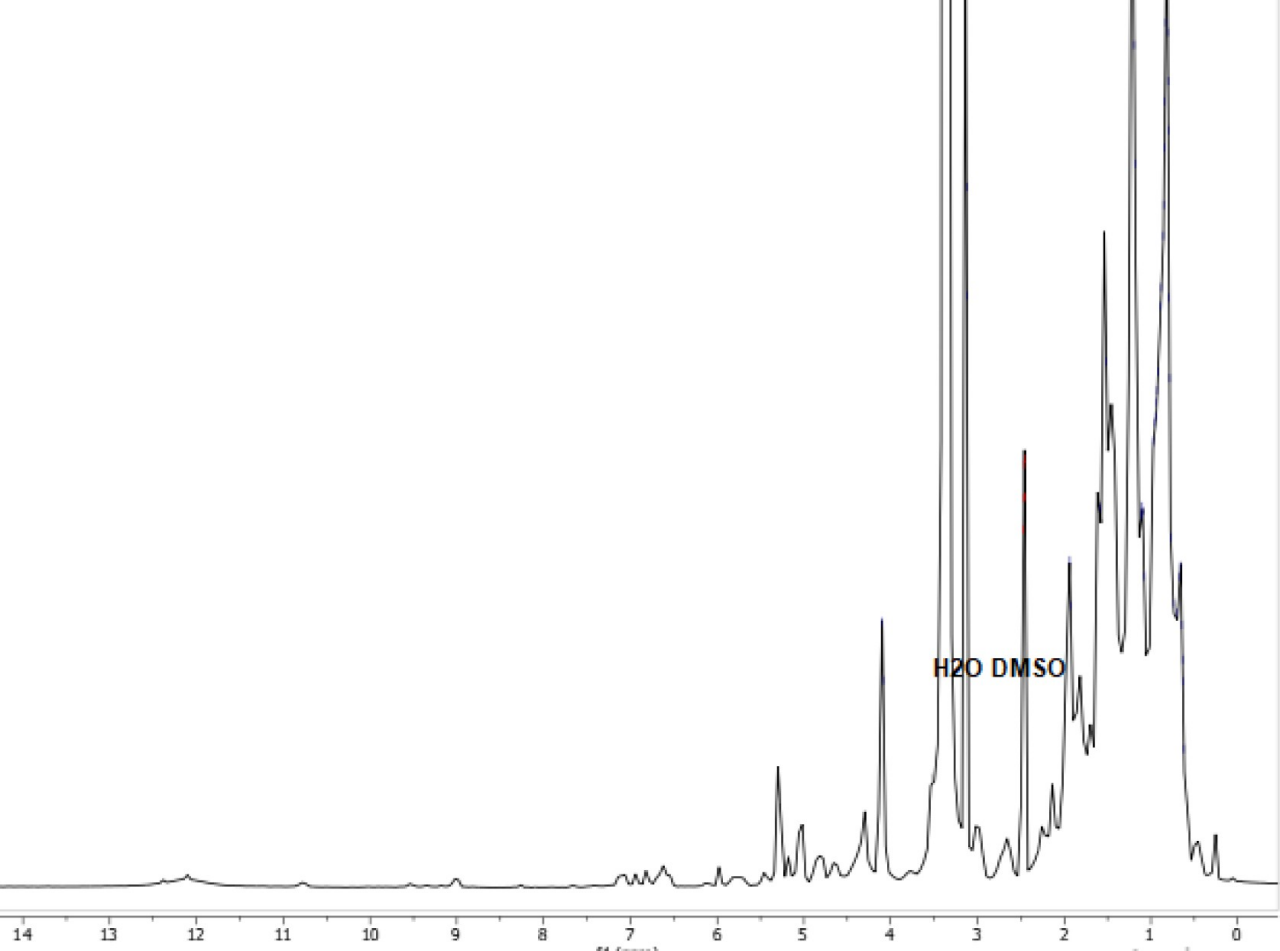
^1^H NMR spectrum of propolis extract using DMSO-d6 solvent.

Based on LCMS/MS analysis, the chromatogram of propolis methanolic extract shows several peaks of bioactive compounds with various retention times. As described in Table 2, there are five main bioactive compounds in propolis methanolic extract that can be detected by LCMS/MS from 8.91–10.40 min of retention time. Additionally, the spectrum of ^1^H NMR containing hundreds of signals is illustrated in Fig 1. The signal shows all the metabolites extracted by the solvent. Further analysis was carried out by comparing the ^1^H NMR with LCMS data using ChemDraw and MestreNova. According to the analysis result, tabulated Table 2 shows evidence of the phytochemical compounds presence in Table 1, such as alisol K 23-acetate and uvariamycin-I as triterpenoids while sugiol and triptobenzene D are diterpenoids. Besides, the evidence of polyphenol is shown by the presence of gomisin K2.

**Table 2:** Bioactive compounds of propolis methanolic extract through LCMS/MS analysis.

No	Retention time (min)	Compounds	Molecular Formula	Detector Count
1	9.64	Alisol K 23-acetate	C_32_H_46_O_6_	1931223
2	9.28	Gomisin K2	C_23_H_30_O_6_	681499
3	9.41	Sugiol	C_20_H_28_O_2_	811168
4	10.40	Uvariamycin-I	C_38_H_70_O_5_	810383
5	8.91	Triptobenzene D	C_20_H_26_O_2_	495563

The ^1^H NMR spectrum shows the presence of alisol K23 acetate through proton signals at chemical shifts of 0.65 (m, 2H), 0.83 (m, 10H), 0.84 (s), 1.20 (m, 8H), 1.41 (s), 1.45 (s), 1.81 (s), 1.51 (s, 1H), 1.53 (s, 2H), 1.60 (s), 1.93 (d, J = 5.7 Hz, 2H), 2.33 (s), 2.34 (p, J = 1.8 Hz, 3H), 2.47 (p, J = 1.8 Hz, 3H), 3.12 (d, 4H), 5.27 (s). Alisol K-23 acetate is one of the triterpenoids that can be isolated from *Alismatis rhizome*. It has been reported to have excellent bioactivity, especially anti-inflammatory, anti-microbial, and antiproliferative properties [30]. Moreover, alisol acetate is known to have anti-diabetic activity by increasing glucose uptake and GLUT4 translocation [31].

The proton signal for gomisin is observed at chemical shifts of 0.83 (m, 7H), 1.20 (m, 6H), 1.51 (s), 2.47 (p, J = 1.8 Hz, 2H), 3.12 (d, J = 2.4 Hz, 3H), 4.09 (d, J = 5.2 Hz, 1H), 4.86 (s), 5.14 (s), 7.42 (s), 7.57 (s), and 7.97 (s) based on the prediction result of ^1^H NMR spectrum. Interestingly, gomisin is a lignin compound that can be isolated from plants of the Schisandraceae family [32]. It is also known to have anti-diabetic activity by increasing AMPK phosphorylation [33], anti-oxidant, anti-tumor, and anti-inflammatory [34]. On the other hand, Fig 1 also shows that the presence of sugiol was indicated by the proton signals at chemical shifts of 0.59 (s), 0.83 (m, 6H), 0.97 (m, 1H), 1.20 (m, 5H), 1.41 (s) 1.60 (s), 1.81 (s), 1.93 (s), 2.46 (p, J = 1.8 Hz, 2H), 2.58 (s), 6.88 (s), 7.5 (s, OH), 7.75 (s). Sugiol is a diterpenoid compound that can be isolated from *Metasequoia glyptostroboides* and the compound exhibited a large number of inhibitory effects on the α-glucosidase and tyrosinase enzymes tested in vitro [34].

Besides, uvariamicin-I was detected at chemical shifts of 0.64 (m, 3H), 0.83 (m, 14H), 0.97 (d, J = 2.7 Hz, 1H), 1.20 (m, 11H), 1.26 (s), 1.41, 1.45 (s), 1.51 (s, 2H), 1.81 (s), 1.93 (d, J = 5.7 Hz, 3H), 2.12, 2.55 (s), 2.99 (s), 3.12 (d, J = 2.4 Hz, 6H), 4.09 (d, J = 5.2 Hz, 2H), and 5.27 (s) ppm. As an acetogenin or natural polyketide product, uvariamicin is commonly found in plants from the Annonaceae family [35]. Fundamentally, acetogenins are recognized as an excellent inhibitory activity against α-amylase and α-glucosidase enzymes [36]. Lastly, Triptobenzene D was measured by proton signals at chemical shifts of 0.83 (m, 6H), 0.97 (m, 1H), 1.10 (m, 1H), 1.20 (m, 5H), 1.41 (s), 1.45 (s), 1.53 (s, 1H), 1.59 (s, 1H), 1.88 (s, CH3), 1.93 (d, J = 5.7 Hz, 1H), 2.46 (pent, J = 1.8 Hz, 2H), 3.12 (d, J = 2.4 Hz, 3H), 4.09 (d, J = 5.2 Hz, 1H), 6.69 (m, 0H), 7.01 (m, 0H), 7.06 (s), dan 7.09 (s). Triptobenzene D is one of the diterpenoids that can be isolated from *Tripterygium wilfordii* [8] and the Celastraceae plant [37].

### Characterization of Pro-ZnO NPs

#### Absorption characteristics of Pro-ZnO NPs

**Fig 2:**
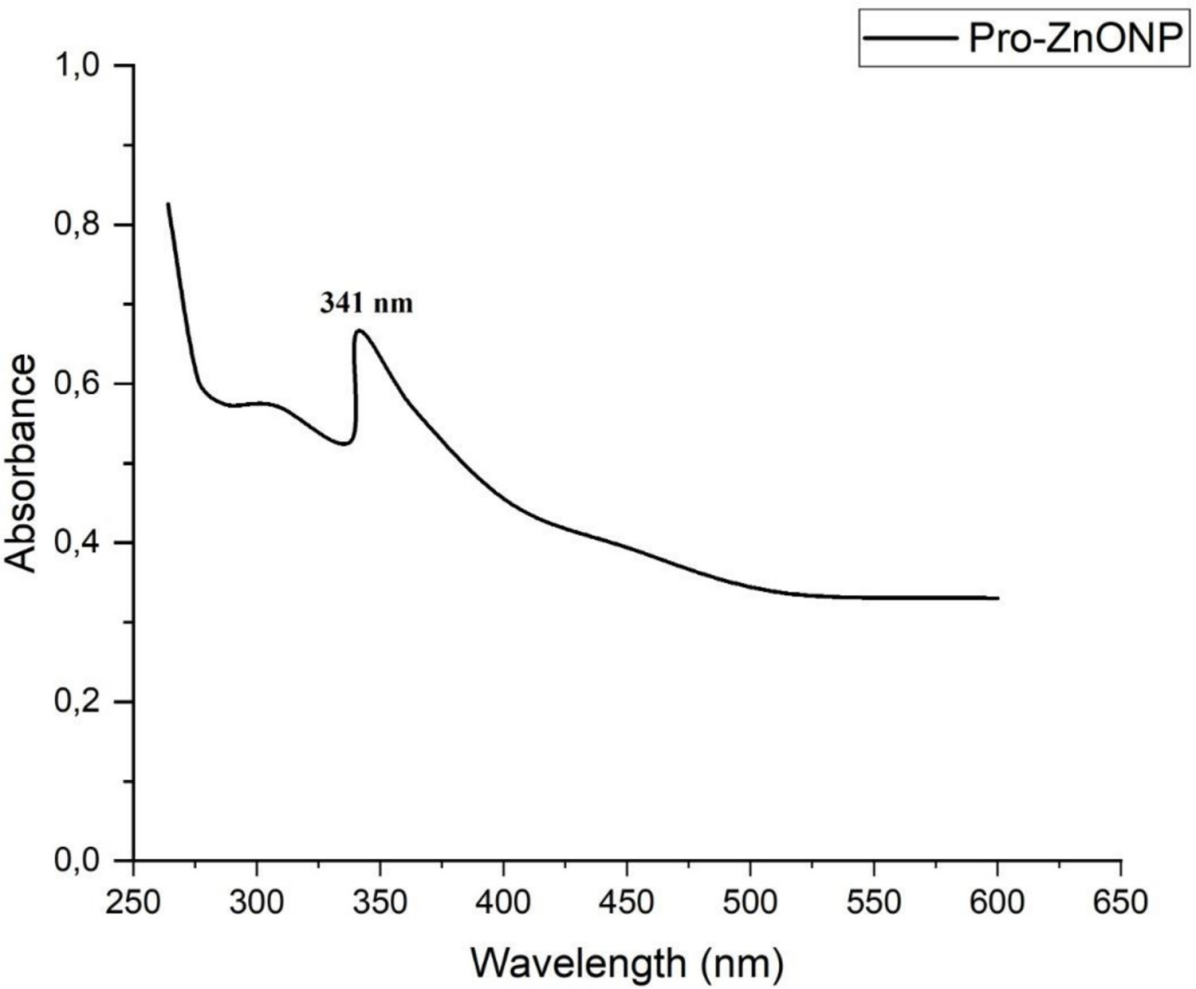
The spectrum of UV-Vis Pro-ZnO NPs.

In order to indicate the absorbability of metallic nanoparticles, their absorbance peak should be measured in correlation with the phenomenon of Surface Plasmon Resonance (SPR) [38]. By emitting the UV-Vis light spectrum at a wavelength (λ) = 260–600 nm, this study revealed the Pro-ZnO NPs absorption band could be noticed at λ = 341 nm, which was typical of ZnO NPs as present in Fig 2. Nonetheless, the change in the color of the solution containing Pro-ZnO NPs and the presence of a characteristic band in the UV-Vis spectrum may be attributed to the SPR phenomenon [39, 40]. Basically, the absorption at λ = 341 nm is in agreement with previous studies reporting that ZnO NPs exhibited a characteristic absorption band between λ = 300–400 nm; therefore, the synthesized nanoparticles were undoubtedly confirmed to be ZnO NPs [33, 41].

Albeit the λ = 341 nm in this research, another possibility may occur when the used materials are different. As such, Vinotha et al. (2019) reported that their ZnO NPs band from *Costus igneus* leaf extract was noted at λ = 365 nm [7]. In addition, Selim et al. (2020) [25] and Jayachandran et al. (2021) [42] revealed the absorption bands of synthesized ZnO-NPs from *Cayratia pedate* and *Deverra tortuosa* were as much as λ = 374 nm and λ = 320 nm, respectively.

#### Morphology and particle size of Pro-ZnO NPs

**Fig 3:**
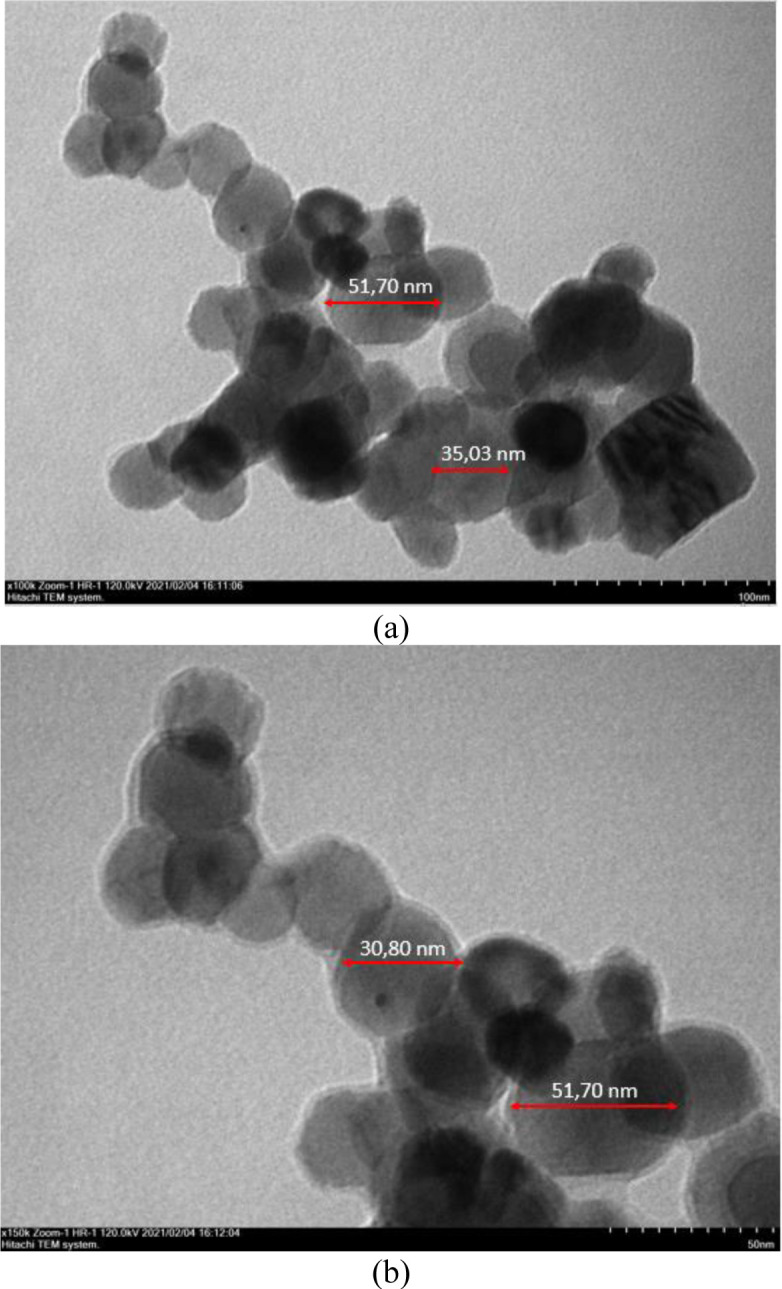
The observations result of nanoparticles using TEM: (a) 100 nm magnification; (b) 50 nm magnification.

The morphology and particle size of synthesized nanoparticles were analyzed using TEM analysis. According to Fig 3, the synthesized Pro-ZnO NPs morphology is recognized as a hexagonal shape with a particle size ranging from 30–50 nm. Straightforwardly, there are several factors that can influence the particle size and morphology such as the pH of the reaction, reactant concentration correlating with the concentration of bioactive compounds of the extract, reaction temperature, and duration of reaction [43].

Despite the diversity of particle sizes, the size is still within the range of other studies. For instance, Barzinjy and Azeez (2020) demonstrated the hexagonal shape of synthesized ZnO NPs using *Eucalyptus globulus Labill*. leaf extract with a size comprised between 27 and 35 nm [26]. Another study also reported the hexagonal shape of synthesized ZnO NPs from *Costius igneus* leaf extract with an average size of 26.55 nm [7]. Furthermore, Jayachandran et al. (2021) also disclosed a hexagonal shape of synthesized ZnO NPs using *Cayratia pedate* leaf extract with an average size of 52.24 nm [42].

### Potential stability of Pro-ZnO NPs

Based on Zeta’s potential analysis, the Pro-ZnO NPs from the biosynthesis possess depicted a negative surface charge of -60.567 mV. This value indicates that the resulting ZnO NPs have excellent stability against particle coagulation [44]. Additionally, the polydispersity index value of Pro-ZnO NP is 0.469, indicating that the particles formed are classified as mid-range monodisperse [45]. Thus, all of the particles can be considered stable and homogenous particles.

### Chemical characteristics of Pro-ZnO NPs

**Fig 4:**
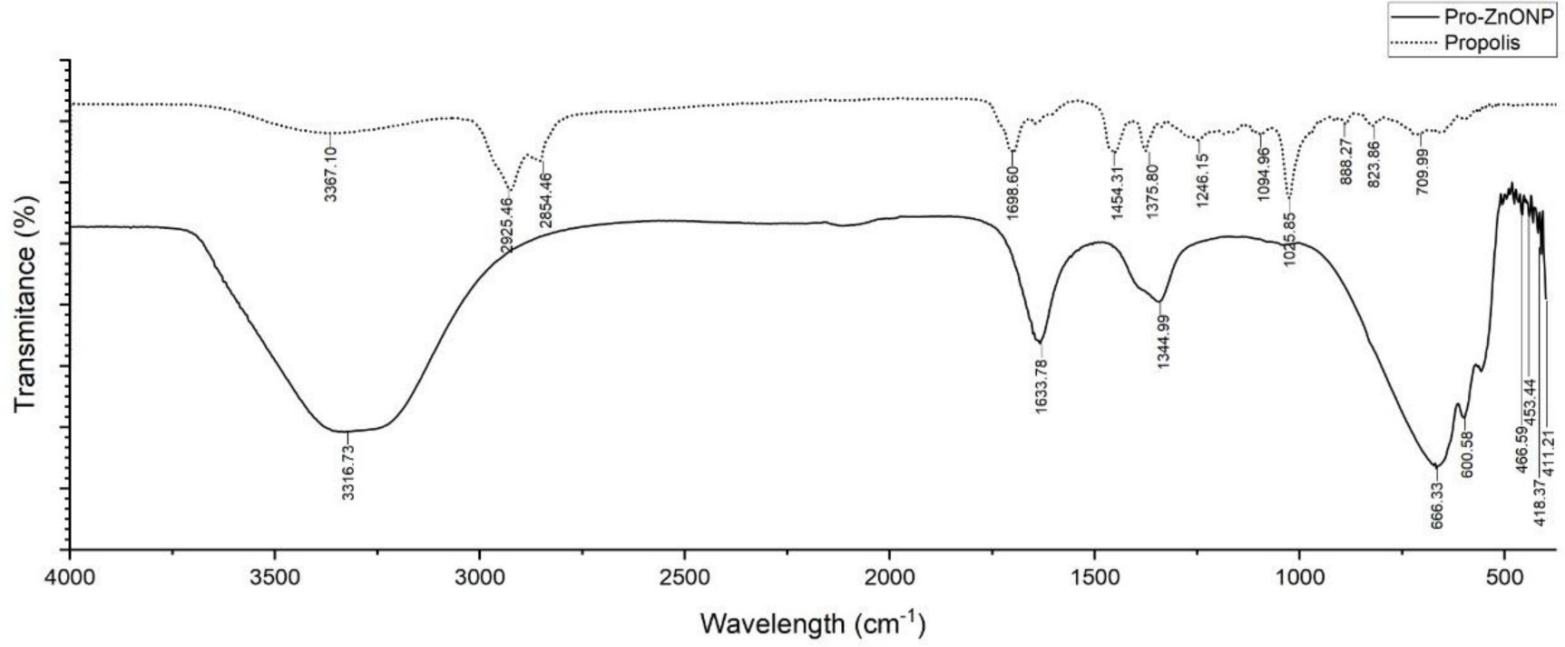
The spectrum of FTIR Pro-ZnO NPs and propolis.

FTIR spectrophotometer was applied to identify functional groups in propolis methanolic extract that contribute to the biosynthesis and stabilization of Pro-ZnO NPs [46]. Based on the FTIR spectrum in Fig 4, it showed a shift at a wavelength (ν) at 3316.73 cm^-1^. This finding becomes evidence of the stretching vibration of O-H functional group of phenol and N-H stretching vibration of amides which are generally found in proteins [47]. Additionally, the existence of those functional groups in the extract is involved in the reduction of zinc into zinc nanoparticles. Interestingly, Daniel and Devi (2019) [48] found compounds that play a role in reducing Zn^2+^ are compounds that are easily soluble in water such as phenolic acids and flavonoid compounds. Another study also reported that protein is involved in the process of reducing Zn^2+^ to ZnO [49]; moreover, the free amine groups of proteins that interact with the zinc surface can affect the stability of ZnO nanoparticles.

Bands at ν = 1698.6 cm^-1^ and ν = 1246.15 cm^-1^ in the FTIR spectrum of propolis extract indicate stretching vibrations of the C = C and C = O groups [50]. The FTIR spectrum of Pro-ZnO NPs also showed similar bands in that area, namely, at wavelengths of ν = 1633.78 cm^-1^ and ν = 1344.99 cm^-1^. Based on Thi et al. (2020), this might be caused by the remaining functional groups such as CO-C, C = O, and C = C in the derivatives of heterocomplex compounds from proteins contained in the propolis extracts [51]. Such functional groups might also serve as a function as capping agents in the synthesis of ZnO nanoparticles and as well as assisting the stabilization of ZnO NPs by forming a layer, covering metal nanoparticles, and preventing nanoparticle agglomeration [25]. These results indicate that propolis bioactive compounds are absorbed on the surface of Pro-ZnO NPs.

On the other hand, the FTIR spectrum of Pro-ZnO NPs also reveals a band between the wavelengths of ν = 411.21 cm^-1^ to ν = 466.58 cm^-1^ which indicates the Zn-O bond’s vibrational strain absorption, confirming that the synthesis method has succeeded in producing ZnO particles [52]. Also, the results are in agreement with ZnO NPs and propolis chemical characteristics that have been previously reported by Barzinjy and Azeez (2020) [26] and Botteon et. al. (2021) [23] based on FTIR analyses. FTIR analyses also indicate the role of chemical compounds in propolis methanolic extract in reducing Zn^2+^ and stabilizing Pro-ZnO NPs and support the idea that biosynthetic nanoparticles are surrounded by a thin layer of phytomolecules including polyphenols, such as flavonoids and tannins, in addition to terpenoids and proteins [23].

### Crystallography of Pro-ZnO NPs

**Fig 5:**
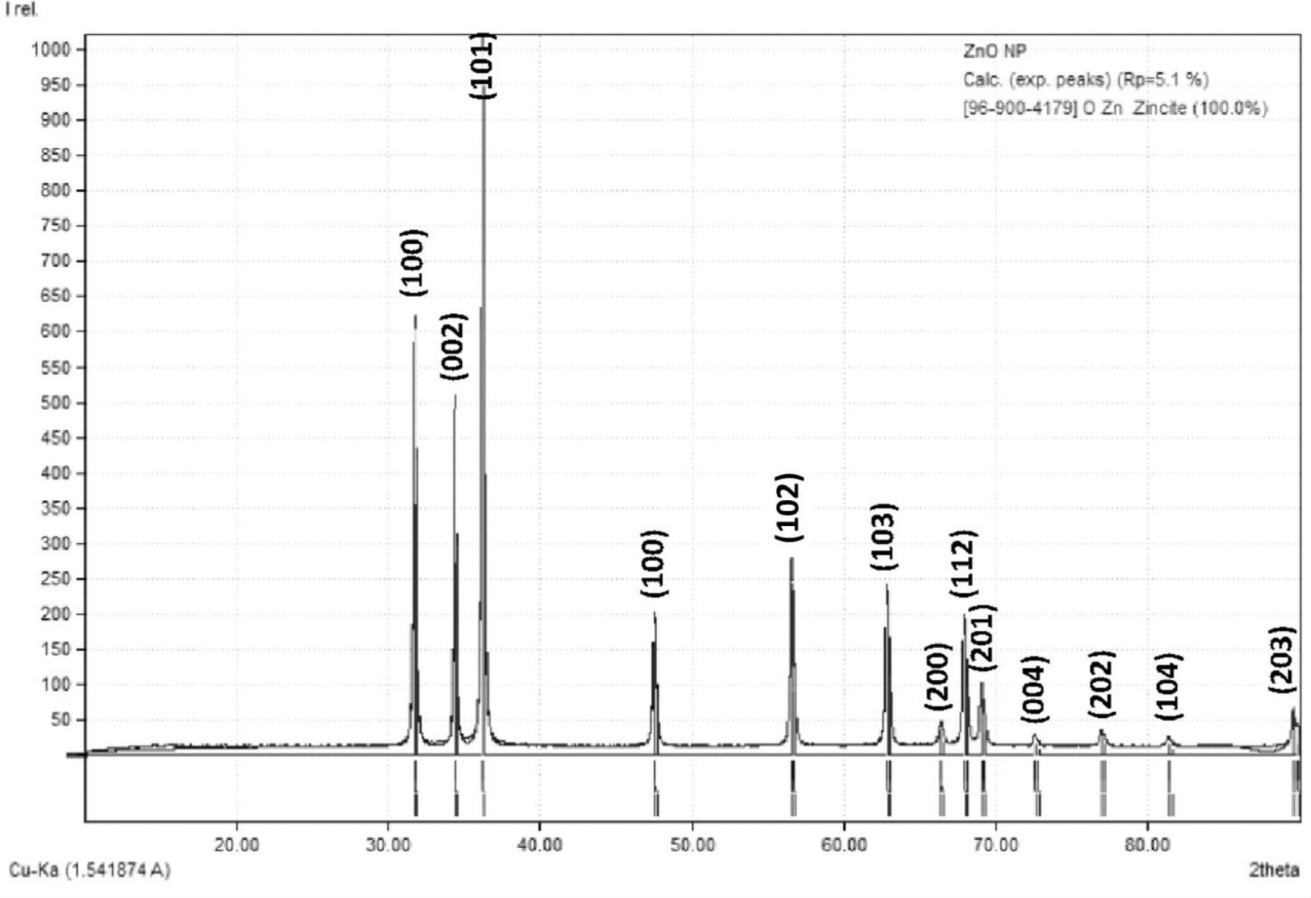
The spectrum of XRD Pro-ZnO NPs.

The diffraction pattern of Pro-ZnO NP was analyzed through X-Ray Diffraction (XRD), and the results are shown in Fig 5. The XRD pattern shows the characteristics of Pro-ZnO NPs, which have a hexagonal wurtzite structure. In addition, compared to the zincite XRD pattern, the synthesized Pro-ZnO NPs showed a synchronous pattern. Moreover, the XRD pattern in this study also has a similar pattern to other studies [11, 22, 53, 54]. Hence, it can be concluded that Pro-ZnO NPs were well synthesized in this study. In detail, the diffraction peaks of the Pro-ZnO NPs sample are at an angle corresponding to the crystal planes of (002), (004), (100), (101), (102), (103), (110), (112), (200), (201) and (202) which conforms to the standard ZnO reference based on the Match3! Program.

According to the Debye-Scherrer equation (S1 Table), the average crystal size of Pro-ZnO NPs is 31.18 nm, confirming the nanometer size of Pro-ZnO NPs. XRD analysis also showed that the degree of crystallinity of Pro-ZnO NPs from the biological synthesis was 82%. Besides, a similar result is shown by the ZnO NPs synthesis study using *Eucalyptus globulus Labill* leaf extract [26]. Basically, a higher level of crystallinity indicates a more orderly arrangement of atoms contained in the material, which in consequence will increase the strength and density of the atomic arrangement [55].

### Antidiabetic activity of Pro-ZnO NPs

#### Inhibitory activity against α-amylase

**Fig 6:**
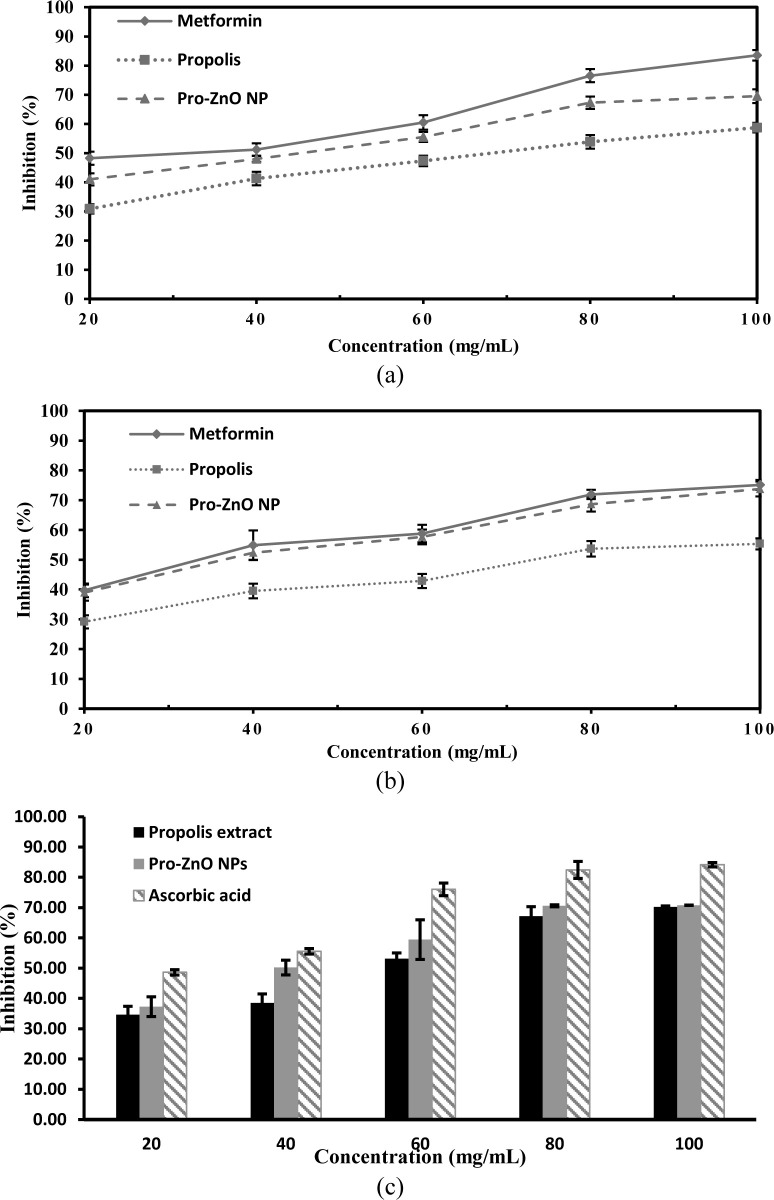
Inhibition rate of (a) Pro-ZnO NPs against α-amylase enzymes; (b) Pro-ZnO NPs against α-glucosidase enzymes; and (c) DPPH radical scavenging activity.

As depicted in Fig 6A, a higher value of α-amylase inhibition was obtained at a higher sample concentration. The results of the propolis inhibition activity against α-amylase ranged from 30.87 (20 mg/mL)– 58.27% (100 mg/mL), while the inhibitory activity of Pro-ZnO NPs ranged from 41.00 (20 mg/mL)– 69.52% (100 mg/mL). On the contrary, the inhibitory activity of metformin fell in the range of 48.24 (20 mg/mL)– 83.52% (100 mg/mL). Accordingly, the inhibitory activity of Pro-ZnO NPs at concentrations of 20, 40, 60, 80, and 100 mg was higher than that of the propolis, and lower when compared to metformin as the positive control at each concentration. The inhibition trend is also indicated by the inhibition effect based on the 50% inhibition concentration (IC50) value of all observed samples, where the IC50 values from highest to lowest are as follows: Metformin (30.77 mg/mL) > Pro-ZnO NPs (43.57 mg/mL) > Propolis (70.54 mg/mL). These data show that Pro-ZnO NPs dan methanol propolis extract has a significant inhibition activity against the α-amylase. Since α-amylase plays an important role in the increase of blood sugar levels, these results hint at the potential of Pro-ZnO NPs in reducing blood sugar levels.

ZnO NPs synthesized from *Costus igneus* extract (Ci-ZnO NPs) showed conformable results. It had higher inhibitory activity against the α-amylase compared to the plant extract. Contrastly, the inhibitory activity was still inferior to the positive control used, namely, metformin [7].

### Inhibitory activity against α-glucosidase

The results of the enzyme inhibition test in Fig 6B shows that the extract of methanol propolis has an inhibitory activity against α-glucosidase of 29.17 (20 mg/mL)– 55.34% (100 mg/mL) with an IC50 value of 77.69 mg/mL. The Pro-ZnO NPs showed the ability to inhibit the activity of the α-glucosidase with an inhibition percentage of 38.97 (20 mg/mL)– 73.78% (100 mg/mL) and an IC50 of 40.69 mg/mL. In contrast, as a positive control, metformin had the most significant inhibitory ability compared to the two samples, with an inhibition percentage of 39.72 (20 mg/mL)– 75.12% (100 mg/mL) and an IC50 of 37.03 mg/mL.

In addition, the in vitro α-glucosidase inhibition test showed that propolis and Pro-ZnO NPs extracts have potential as antidiabetics. However, the inhibitory activity of the synthesized Pro-ZnO NPs was still higher than the propolis methanolic extract and comparable with the inhibitory activity of metformin as the positive control. As a validation, similar results were also shown by ZnO NPs from green synthesis using *Costus igneus* extract (Ci-ZnO NPs). The synthesized ZnO NPs had a higher inhibitory activity on α-amylase and α-glucosidase when compared to the plant extract. Nonetheless, it was still lower than metformin used as a positive control [5].

Notably, both zinc (Zn^2+^) [56] and propolis [14] have an anti-diabetic effect. Hence, the combination of zinc (Zn^2+^) with propolis extract might produce a synergistic interaction in increasing the antidiabetic action of Pro-ZnO NPs. This is due to the presence of proteins and amino acids along with other phytochemical constituents that bind to zinc.

### Antioxidant activity of Pro-ZnO NPs

The antioxidant activity of Pro-ZnO NPs was determined using DPPH as a free radical scavenging assay. DPPH is characterized as a stable free radical by virtue of the delocalization of free electrons over the molecule so that the molecules do not dimerize and give rise to the deep purple-colored solution. DPPH is characterized by absorption at around λ = 520 nm in ethanol or methanol solution. Upon mixing the free radical DPPH with an antioxidant substance, the substance can donate a hydrogen atom to DPPH and result in a yellow-colored solution containing reduced DPPH [47, 57]. The result of the antioxidant activity of the biosynthesized Pro-ZnO NPs using DPPH free radical scavenging assay is shown in Fig 6C. The inhibitory activity against the DPPH free radical of ascorbic acid, Pro-ZnO NPs, and propolis extract revealed IC50 values of 20.46, 42.49, and 54.52 mg/mL, respectively.

Each sample experiences a growth of scavenging activity with increasing concentration. The biosynthesized Pro-ZnO NPs present higher antioxidant activity than propolis extracts with the highest concentration at 70.76% at 100 mg/mL. Nonetheless, the activity was lower when compared to the scavenging activity of ascorbic acid as a positive control. The antioxidant activity of biosynthesized Pro-ZnO NPs might be due to the electrostatic attraction between (ZnO → Zn^2+^ + O^2-^) and bioactive compounds of propolis extract. The phytochemical compounds associated with propolis extracts including phenolic, flavonoids, terpenoids, and tannins can scavenge and quench free radical molecules simultaneously giving rise to its antioxidant activity [47].

Remarkably, the increase in antioxidant activity of the biosynthesized Pro-ZnO NPs when compared to propolis extract might be a result of metal ions that are present in the nanoparticles. Pękal and Pyrzynska (2015) [56] demonstrate that the complex of metal ions (Al, Cu, and Zn) and flavonoids, as well as quercetin and epigallocatechin (EGCG), showed a higher efficiency on DPPH radical scavenging activity rather than the group without added metal. Furthermore, those metal-flavonoid complexes have much stronger free radical scavenging activity properties than the free flavonoids due to the metal ion chelation ability possessed by those compounds [58, 59]. Consequently, it seems that the metal ions including zinc could influence the stability of particular phytochemical compounds such as phenol derivatives and flavonoids, which can chelate metal ions.

## Conclusions

The synthesis of zinc oxide nanoparticles using methanol propolis extract (Pro-ZnO NPs) through a biological synthesis method was successfully conducted. Obtained Pro-ZnO NPs were proven as nanoparticles with hexagonal wurtzite shape. In brief, Pro-ZnO NPs’ inhibitory activity against α-amylase and α-glucosidase has been verified by a high inhibition rate. Then, the Pro-ZnO NPs can then be utilized as a potential natural antidiabetic agent. Hence, this study could contribute a novel pathway to produce antidiabetic agents by using natural resources as well as propolis.

## Supporting information

S1 Table Calculation of the particle size of Pro-ZnO NPs using the Debye–Scherrer equation. (DOCX)
