# Supplementary material for: Synthesis of zinc oxide nanoparticles using methanol propolis extract (Pro-ZnO NPs) as antidiabetic and antioxidant
Source: PLoS One. 2023 Jul 25;18(7):e0289125. doi: 10.1371/journal.pone.0289125 (PMC10368249; doi:10.1371/journal.pone.0289125)
Supplement: S1 Table — (DOCX) [file pone.0289125.s001.docx]

**Table S1. Calculation of the particle size of Pro-ZnO NPs using the Debye–Scherrer equation**

| Peak Number | h k l | Position  (°2Th) | FWHM  (°2Th) | Crystal Size (nm) |
| --- | --- | --- | --- | --- |
| 1 | 100 | 31.76 | 0.203 | 37.64093677 |
| 2 | 002 | 34.41 | 0.188 | 40.36597001 |
| 3 | 101 | 36.24 | 0.2144 | 35.2159967 |
| 4 | 100 | 47.54 | 0.2564 | 28.3556226 |
| 5 | 002 | 56.56 | 0.2186 | 32.00402939 |
| 6 | 110 | 56.72 | 0.1954 | 35.77696355 |
| 7 | 103 | 62.82 | 0.2206 | 30.73495792 |
| 8 | 103 | 63.00 | 0.1887 | 35.89623683 |
| 9 | 200 | 66.38 | 0.3321 | 20.01883968 |
| 10 | 112 | 67.91 | 0.2287 | 28.8132973 |
| 11 | 112 | 68.11 | 0.2122 | 31.017185 |
| 12 | 201 | 69.06 | 0.248 | 26.39007941 |
| 13 | 201 | 69.26 | 0.2103 | 31.08354873 |
| 14 | 004 | 72.53 | 0.1768 | 36.22963223 |
| 15 | 004 | 72.75 | 0.1622 | 39.43505141 |
| 16 | 202 | 76.92 | 0.2614 | 23.79769102 |
| 17 | 202 | 77.16 | 0.1966 | 31.58878022 |
| 18 | 104 | 81.38 | 0.3823 | 15.75658474 |
| 19 | 203 | 89.58 | 0.2412 | 23.37484214 |
| 20 | 203 | 89.85 | 0.1404 | 40.06274257 |
|  |  |  | Average | 31.17794941 |
|  |  |  | Std.Dev | 6.781598998 |
